# The Minimally Invasive Bipolar Fixation for Pediatric Spinal Deformities: A Narrative Review

**DOI:** 10.3390/children11020228

**Published:** 2024-02-09

**Authors:** Lotfi Miladi, Federico Solla, Mathilde Gaume

**Affiliations:** 1 Pediatric Orthopedic Surgery Department, Necker Hospital, Assistance Publique des Hopitaux de Paris (APHP), University of Paris-Cité, 75105 Paris, France; l.miladi@aphp.fr; 2 Pediatric Orthopaedic Surgery, Lenval University Children’s Hospital, 06200 Nice, France; federico.solla@hpu.lenval.com; 3 University Institute for Spine Surgery, Armand Trousseau Hospital, Sorbonne University, 75012 Paris, France

**Keywords:** neuromuscular scoliosis, fusionless technique, minimally invasive spinal fixation, bipolar technique, self-expanding growing rod, spine, scoliosis, children

## Abstract

Growing rod techniques are increasingly used for early-onset scoliosis in children. Unfortunately, they are associated with many complications, particularly neuromuscular scoliosis, favored by the poor general condition of these patients and the fragility of their osteoporotic bones. Furthermore, these interventions are often iterative and usually followed by vertebral fusion at the end of growth. This is a review of the literature on a recent fusionless technique, minimally invasive bipolar fixation, which is more stable than the traditional growing rod techniques and less aggressive than vertebral arthrodesis. It allows the avoidance of arthrodesis, owing to the solidity of the construct and the stability of the results, leading to progressive spinal stiffening that occurs over time. The results of this technique have been published with a long follow-up period and have confirmed that it can completely replace posterior vertebral arthrodesis, especially in the most complicated scoliosis. Because it preserves growth, this technique should be recommended for early-onset scoliosis before the age of 10 years. The use of a self-expanding rod can avoid the need for repeated surgery, thereby reducing the risk of complications and the overall cost of treatment.

## 1. Introduction

Surgical treatment of spinal deformities has progressed significantly over the last 30 years. The introduction of Cotrel–Dubousset (CD) segmental instrumentation in 1983 revolutionized the surgical treatment of spinal deformities [1]. The CD instrumentation allowed excellent posterior vertebral fusion and three-dimensional correction of scoliosis, while the solidity of the implants meant that the patients did not need to wear casts or braces after surgery.

The original CD instrumentation has inspired many surgeons and companies in the last 20 years. Therefore, many varieties of spinal instrumentation have emerged, with technical improvements in both implants and ancillaries, which have become increasingly more efficient and now allow excellent correction. As a result, surgeons are increasingly using multilevel segmental fixation for spinal fusion [2].

Spinal deformity begins early in neuromuscular diseases and usually progresses despite bracing, requiring early surgery that may limit thoracic and spinal growth and reduce respiratory function. Posterior fusion is the riskiest surgical treatment for neuromuscular scoliosis. In a series of 8975 patients undergoing orthopedic surgery, posterior spinal fusion was the only procedure complicated by death within the first months [3].

Many types of fusionless techniques have been used over the last 20 years, but they have suffered from complications, especially neuromuscular scoliosis [4,5,6,7,8,9,10,11,12,13]. For this reason, we have developed a minimally invasive bipolar fixation (MIBF) with the aim of offering a less aggressive alternative to posterior spinal fusion and a stronger stability than traditional fusionless techniques. The first recipients of this technique were patients with neuromuscular and syndromic diseases.

Vertebral fusion is not suitable for young children with low skeletal maturity and progressive spinal deformity, despite conservative management because arthrodesis stops the remaining vertebral growth with concomitant effects on lung development [14]. In these cases, other types of early surgery can be used, such as fusionless instrumentation, which was developed in the 1990s [15]. The aim of early surgery is to ensure good correction of spinal deformities, while preserving spinal growth. These fusionless techniques use costal or vertebral fixations that allow different types of constructs, commonly referred to as traditional growing rods or “TGR” [4,5,6,7]. They are used worldwide.

Unfortunately, these early surgeries have proven to be a source of many mechanical complications due to the bone fragility of young patients, as well as errors in technique and/or surgical strategy on the part of the surgeon [5,6,7]. These early and repeated surgeries induce fibrosis and early spontaneous fusion of the spine, leading to premature arthrodesis [8,9,10,11,12].

In addition, posterior fusion for neuromuscular scoliosis has a high complication rate, due to the fragile population and poor bone quality [3,13,16,17,18,19,20,21]. 

The aim of this narrative review was to update the MIBF for pediatric scoliosis.

## 2. Relevant Sections

### 2.1. The Concept of Bipolar Fixation

In the search for a surgical technique that reduces the rate of complications, we progressively improved the TGR technique and started using fusionless MIBF for neuromuscular scoliosis in 2010 [22].

The concept of this technique is based on a solid and definitive construct that allows for good correction and stabilization of spinal deformity, avoiding early auto-fusion and fibrosis. The spinal and thoracic growth is preserved, and progressive spinal ankylosis occurs after many years, avoiding the need for a final fusion at the end of growth. 

In fact, the MIBF method acts as a delayed fusion because the final result is complete spinal ankylosis of the spine that occurs progressively many years after the initial surgery, owing to the presence of a rigid construct in the patient’s back [23]. Contrary to the TGR technique, in which autofusion and fibrosis occur in the first years after the index surgery, induced by multiple rod lengthening performed in the intermediate area, the MIBF technique completely preserves the intermediate area and avoids early fibrosis and spontaneous fusion. Many years before the advent of autofusion, the surgeon can improve the correction of residual deformities and follow skeletal growth because of the rod lengthening procedures performed on demand outside the intermediate area.

In the MIBF method, if the conditions of the patient and the hospital allow it, we recommend the preparation of severe and rigid spinal deformities by preoperative halo-gravity traction for 4 to 6 weeks, Stagnara casting, or other external stretching techniques. This preparation has been shown to improve correction and reduce the risk of mechanical and neurological complications [24]. 

The MIBF method has been improved over time and has, in the last 12 years, become a real alternative to posterior vertebral arthrodesis for juvenile and immature (Risser 0) neuromuscular scoliosis (including cerebral palsy, spinal muscular atrophy, flaccid paraplegia, Rett syndrome, and muscular dystrophy) and for other types of complex and progressive scoliosis [25].

**Figure 1:**
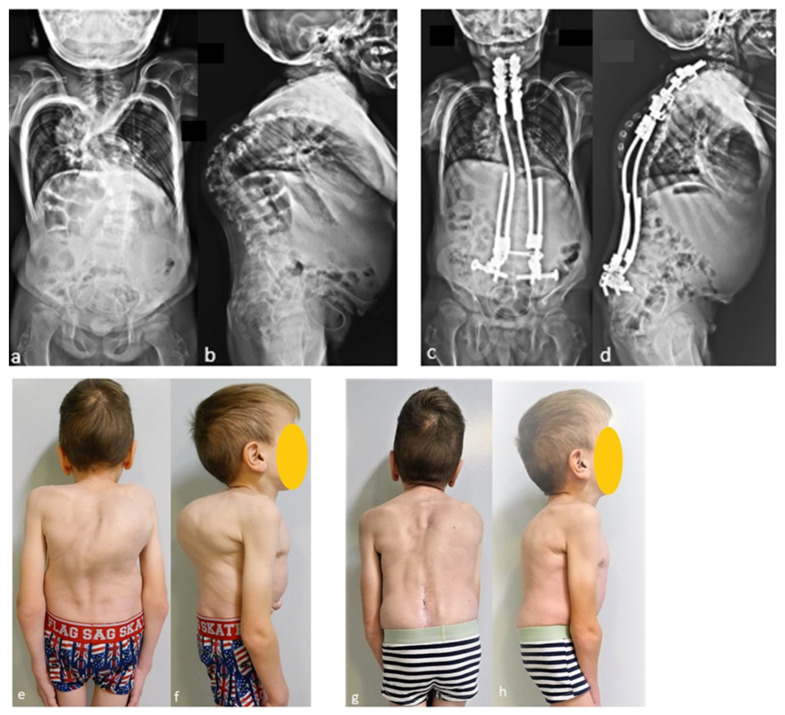
(**a**–**d**) Preoperative radiographs and pictures of a 10-year-old boy with kyphosis due to Morquio syndrome; (**e**–**h**) 2 year postoperative radiographs and pictures.

The case shown in the first figure is a boy with Morquio syndrome with severe kyphosis who underwent MIBF extended to the pelvis after 4 weeks of preoperative halo-gravity traction (Figure 1).

### 2.2. The Surgical Technique

The patient lies prone on a spinal operating table, with traction on the lower limbs, and sensorimotor-evoked potential control, if necessary.

#### 2.2.1. Iliosacral Screws

**Figure 2:**
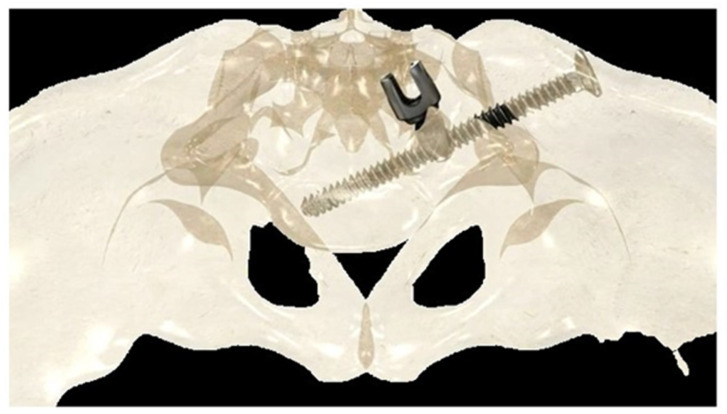
Drawing showing the bony path of the iliosacral screws passing through the ring of the iliosacral connectors.

If the construct included the pelvis, an initial short midline incision was made at the lumbosacral junction. A Wiltse intramuscular paramedian approach was used to expose the lumbosacral joint and posterior cortex of the sacrum on each side. A small hole was then created in the sacral cortex, lateral to the articular process of S1, and above the first posterior sacral foramen. An iliosacral connector is inserted into the sacral hole. This connector has a deep ring, into which an iliosacral screw is introduced percutaneously using a bespoke jig, following an oblique posteroanterior direction that avoids the spinal canal, starting from the iliac crest and ending in the body of S1 (Figure 2). The open and polyaxial iliosacral connector allows an easy and secure connection of the rod to the iliosacral screw. The connector has a low profile and is located in a deep position, allowing its use in very young and skinny patients with a body weight of less than 15 kg.

The deep intraosseous direction of the iliosacral screw provides strong and stable pelvic fixation that can be used in all conditions, especially in neuromuscular patients with very osteoporotic bone [26,27].

#### 2.2.2. Lumbar Fixation

If the construct does not include the pelvis, for example, in walking patients, distal fixation is achieved with two or three pairs of pedicle screws in the lumbar vertebrae, placed through a midline skin incision, and a para-median Wiltse intramuscular approach used for pelvic fixation.

#### 2.2.3. Thoracic Fixation

A second short midline incision is made at the proximal thoracic spine, usually starting at the first (or second) thoracic vertebra. Subperiosteal exposure of the lamina from T1 to T4 or T5 was performed, and then two pedicle supra-laminar hook claws were placed at T1–T2 and T4–T5 on each side. This type of proximal hook claw fixation is a strong fixation technique that provides high resistance to pull-out forces, even in kyphotic deformities. Other types of fixations are also described with pedicle–transverse claws, usually between T2 or T3, and T6 or T7. Then, two small, pre-shaped rods were inserted into the hooks and fixed together by two cross-links to realize a strong proximal frame construct. A combination of hooks and screws, or all screws, is also possible depending on the surgeon’s experience.

#### 2.2.4. Connections and Correction

An intermediate rod is then inserted into the concavity of the main curve and connected to the proximal rod by a cylindrical connector and is connected to a small distal and lateral rod by a side-to-side closed domino. The distal rod is then inserted into the distal anchors, into either the iliosacral connector or the lumbar pedicle screws.

It is also very important to perform adequate rod contouring prior to insertion, to avoid any constraints that may weaken the bone anchors.

Gentle progressive distraction maneuvers are performed on the domino connecting the intermediate and distal rods, correcting the spinal deformity in the frontal plane, which may be supplemented by an in situ rod bending maneuver, to improve the sagittal plane correction if necessary. The intermediate and distal rods on the convex side are generally inserted without additional maneuvers.

**Figure 3:**
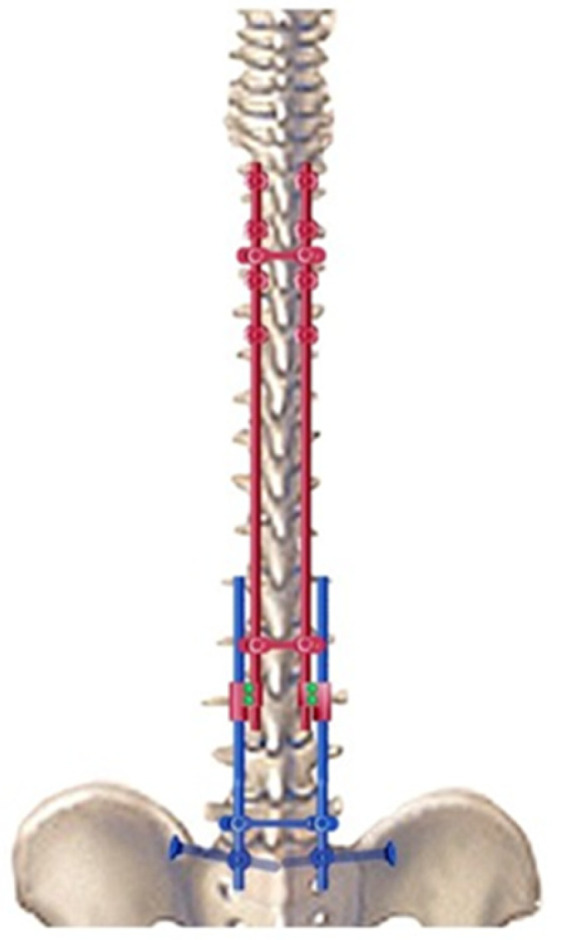
Drawing of the bipolar sliding construct with pelvic fixation.

In neuromuscular scoliosis, if the pelvis remains oblique despite distraction on the concave side, a compression maneuver can be performed on the convex side to improve or complete correction. Finally, a third cross-link device was placed near the iliosacral connectors to provide solid and stable distal fixation for the long construct. This sliding construct allows for future lengthening of the rods, if required (Figure 3).

#### 2.2.5. Postoperative Care and Follow Up

In the postoperative period, patients are able to sit or stand without needing a brace 1 to 2 days after surgery and are discharged 5 to 7 days later.

**Figure 4:**
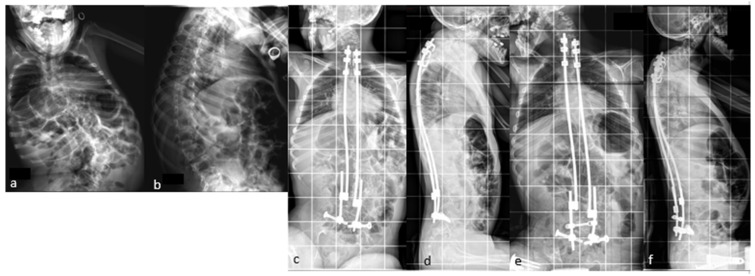

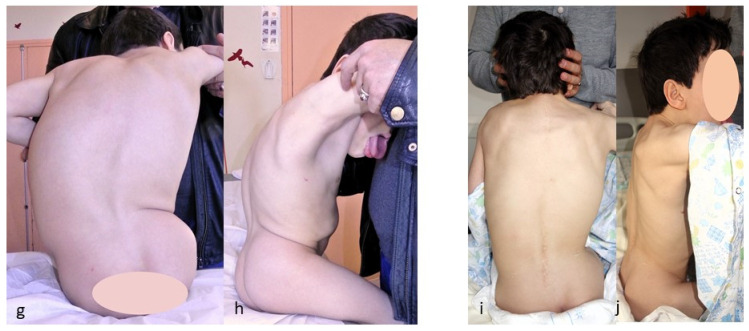
(**a**,**b**) Preoperative radiographs of a 12-year-old boy with cerebral palsy; (**c**,**d**) postoperative radiographs; (**e**,**f**) Radiographs after rod lengthening; (**g**–**j**) initial and 6-year postoperative clinical pictures.

If necessary, additional rod lengthening can be performed one–two years after the initial surgery to correct the residual curve or pelvic obliquity, or to accommodate growth in a young skeletally immature patient (Figure 4).

## 3. Results in Recently Published Literature

The literature search allows individuate twelve articles on fusionless MIBF (Table 1). 

A series of the first 100 patients with neuromuscular scoliosis underwent MIBF at an average age of 11.5 years. The results were published in 2021, with a minimum follow up of 5 years [25]. The mean correction rates at the last follow up were 67% for spinal deformity and 83% for pelvic obliquity. This degree of correction of pelvic obliquity was achieved by an asymmetric additional rod-lengthening procedure, as shown in Figure 4. The overall complication rate in this series was 23%, of which 16% were infectious. The majority of cases were cerebral palsy, and five cases had spina bifida as the etiology. A total of five cases of rod fracture were reported in the first series. Vertebral arthrodesis was not performed for any patient in this study.

This technique was used in another series of 59 cases of scoliosis in spinal muscular atrophy (SMA) with the mean age at surgery of 11 years [28]. The mean preoperative body weight was 30.6 kg and 35.5 kg at the last follow up, with a mean gain of 6.1 kg at 3 years postoperatively. All of the patients had previously undergone conservative treatment with bracing. A total of ten patients required halo traction prior to surgery for stiff curves with Cobb angle > 90°. The mean follow-up durations were 5.2 years. The initial Cobb angle was 80°, which reduced to 42° at the last follow up, with a correction of 48%. The preoperative pelvic obliquity was 24° and 6° at the last follow up, with a 76% correction rate. There were five cases of deep infection and eight cases of mechanical complications, resulting in six unplanned surgeries. The mechanical complications were proximal hook dislodgement, one proximal rod, one iliosacral connector prominence, and four cases of iliosacral screw malposition. No rod fractures were observed in this study. Of the patients and their families, 91.5% were satisfied with the procedure. None of the patients in this series underwent arthrodesis at the end of their growth period.

A study compared perioperative complications between arthrodesis and MIBF in 140 neuromuscular scoliosis patients [29]. There were 140 patients (70 M and 70 F) who underwent surgery at a mean age of 13.2 years: in total, seventy-five were treated with posterior spinal fusion and sixty-five with MIBF. The coronal corrections were similar between the groups, and in the MIBF group, the Cobb angle was reduced from 89° to 43°. Pelvic obliquity was also reduced from 29° to 17°. In the posterior fusion group, the Cobb angle decreased from 76° to 38° and pelvic obliquity was reduced from 24° to 15°. More patients in the fusion group (81%) required a blood transfusion than those in the minimally invasive group (23%). During the postoperative ICU stay, the fusion group had more complications, such as infection (35% vs. 13%) and hemodynamic complications (22% vs. 6%). The average length of stay in the ICU was 5 days in the fusion group and 4 days in the minimally invasive group. Thus, in this series, the overall complication rate was lower for MIBF procedures (40%) than for posterior spinal fusion procedures (76%).

A similar study was carried out by Vergillos-Luna et al. [30], comparing 48 standard fusion surgeries and 41 MIBF in neuromuscular scoliosis patients from two centers, which was different from that of the previous studies. MIBF was associated with fewer transfusions (27 vs. 73%), less estimated blood loss, fewer major complications (32 vs. 52%), and less unplanned surgery (15 vs. 39%). 

To complete the drawing, in an adult series by Wolff et al., 15 patients who underwent conventional instrumentation with pedicle screws, hooks at the top, and iliac screws in the pelvis were compared to 31 patients who underwent minimally invasive surgery. Neither correction nor surgical complications differed between the two groups at 3 years. However, blood loss and medical complications were 50% lower in the minimally invasive fusionless surgery group [31].

**Table 1:** Summary of main MIBF published papers.

	No of Patients	Follow-Up, Mean	Pelvic Obliquity Correction	Cobb Angle Correction	Mechanical and Infectious Complications
Miladi et al. Spine, 2018 [22]	100	3 years	29° to 5°	89° to 35°	26%
Gaume et al. Spine, 2021 [25]	83	6.5 years	29° to 7°	89° to 35°	31.3%
Gaume et al. JPO, 2021 [28]	59	5.2 years	24° to 6°	79° to 41°	15%
Gaume et al. JBJS open access, 2021 [32]	21	3 years	20° to 8°	66° to 32°	24%
Gaume et al. AOTS, 2023 [29]	65	N/A	29° to 17°	89° to 43°	20%
Gaume et al. AOTS, 2023 [33]	167	6.4 years	20° to 5°	75° to 36°	N/A
Gaume et al. Eur Spine J, 2023 [23]	19	6 years	N/A	89° to 35°	N/A
Vergillos-luna et al. Clin Spine Surg, 2023 [30]	41	2 years	16° to 10°	70° to 38°	32%
Wolff et al. Global Spine J, 2023 [31]	31	3 years	18° to7°	70° to 36°	29%

N/A = not available.

## 4. Discussion

At the beginning of our experience, MIBF was used as an alternative to traditional growing rods (TGR) to reduce the complication rate while waiting for spinal fusion at skeletal maturity. However, the stability of the correction and the reduced complication rate allowed us to avoid spinal fusion, especially when we noticed spontaneous fusion around the implants during the revision surgery for rod expansion. The records of the first 100 neuromuscular scoliosis patients operated on using MIBF were reviewed at a follow up of 6.5-years. The results were stable, even in patients who reached skeletal maturity. In older patients, no fusion was required at a follow up of >12 years [25].

The concept of autofusion is old and has been reported by several authors who performed subcutaneous rodding without fusion, such as in 1984 using the Harrington rod [34] and Mardjetko et al. with Luqué rods [35,36,37,38,39]. More recently, Cahill reported an 89% autofusion rate in a series of TGR [8], and Jain noted progressive spinal ankylosis after fusionless surgery, which may allow the avoidance of arthrodesis in patients with satisfactory correction and stable constructs [38].

In TGR, autofusion occurs early because of the iterative surgical approach in the intermediate area, leading to the “law of diminishing return” [10] and necessitating a final fusion to improve the correction of the residual deformity. 

Magnetically controlled growing rods have been developed as an alternative to TGR to avoid iterative surgeries for rod lengthening [39,40]. However, they also have a reduced ability to lengthen over subsequent lengthening [41]. However, the complication rate remains high. In a recent NMS series, 33% of patients had at least one complication during follow up, with 44% of mechanical complications and 28% of patients undergoing final fusion at skeletal maturity [42].

Conversely, in the MIBF technique, autofusion occurs progressively for many years after the initial surgery. Delayed autofusion induced by MIBF was confirmed by a CT scan study of ten patients operated on with this technique. Induced fusion was present in 93% of the patients at a mean follow up of 10.7 years [23]. A shear wave elastography analysis of the lumbar annulus fibrosus also showed that neuromuscular scoliosis patients treated with MIBF had a significant increase in disc stiffness at the end of growth [43].

The neuromuscular population is fragile, with multiple comorbidities and a very osteoporotic bone, which explains the higher complication rate (24–75%) than in congenital (10.6%) and idiopathic (6.3%) scoliosis [3]. Another author, Connie Poe-Kochert, reported an average of 1.5 complications per patient after spinal fusion in patients treated with growing rods [44]. Therefore, avoiding arthrodesis with MIBF is useful in neuromuscular patients because the construct is solid enough to be definitive.

**Figure 5:**
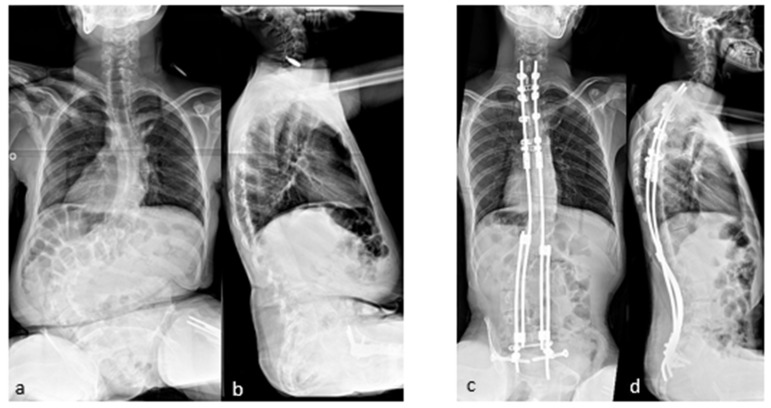
(**a**–**d**) Pre- and postoperative radiographs of 14-year-old patient with cerebral palsy who underwent surgery with a reinforced lumbar four-rod construct.

This technique can be considered as a real alternative to arthrodesis in this population, as compared to other techniques that report 16.6% rod breakage in neuromuscular series [16], 15–42% with TGR in early-onset scoliosis [1,2,3,4,5,6,7,8,9,10,11,12,13,14], and 70% in a cerebral palsy series [17]. It provides satisfactory correction results that remain stable until skeletal maturity, with a reduced rate of mechanical complications (5% of rod fractures in our initial series at 5 years follow up) [25]. Patients at risk of rod fractures include ambulatory patients, those with dystonia, those with a higher BMI, or those with excessive kyphosis. Currently, this complication can be avoided using reinforced four-rod constructs in the lumbar region or throughout the spine in patients with any of these risk factors (Figure 5).

Compared to other series [21], the lower rate of mechanical complications with the MIBF technique may be explained by the solidity of proximal fixation with a double hook-claw construct, combined with meticulous proximal rod bending and pelvic fixation with iliosacral screws distally.

Infectious complications in our series were lower than those in TGR in cerebral palsy patients (30%) [17] or in the spinal muscular atrophy series [18], probably due to the lower number of iterative surgeries in the MIBF method than in the TGR technique [9].

In the series of 59 spinal muscular atrophy scoliosis [28], operated on at a mean age of 11 years at first surgery, the most important finding was that clinical and radiological outcome scores improved significantly after minimally invasive fusionless surgery with a reduced complication rate. These results remained consistent over a long follow-up period, and arthrodesis was not required in any patient. Remarkably, there was no deterioration in respiratory function after MIBF.

This technique improved the patients’ quality of life and satisfaction by 91.5% by avoiding postoperative bracing. Negative satisfaction with the procedure in 5/59 patients was mainly due to complications or the inability to avoid bracing due to a previous lack of head control. Arthrodesis was not performed, although some patients had been followed up for almost 10 years because of the stable construct, resulting in gradual and delayed spinal ankylosis with visible degeneration of disc spaces and articular processes on radiographs and CT.

**Figure 6:**
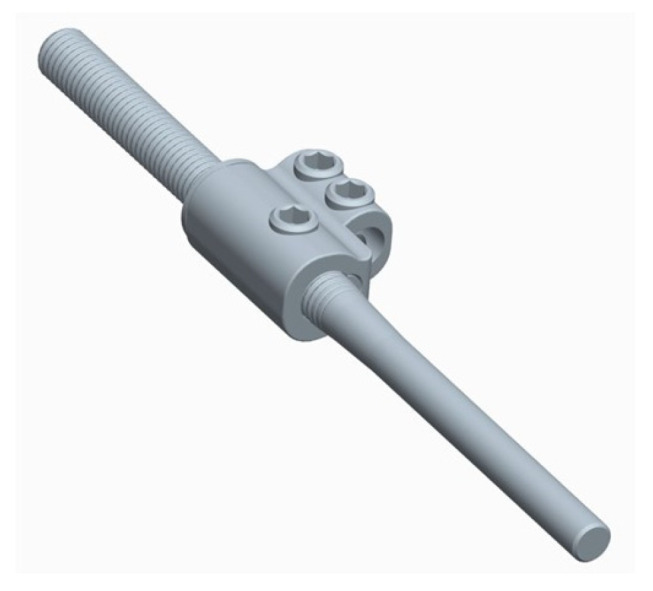
Illustration of a self-expanding rod.

Like all traditional growing rods, conventional MIBF still requires repeated surgery to perform iterative rod-lengthening procedures. This leads to an increased risk of infectious complications. This led to the development of a new self-expanding rod (Nemost rod; Euros Company, La Ciotat, France), which consists of a rod with a notched section representing the lengthening reserve on which a domino slides in one direction, allowing a progressive lengthening of the construct (Figure 6). This expansion of the rod can occur in two ways: passively during the patient’s daily movements, during bony growth of the spine, or actively during symmetric or asymmetric axial traction exercises of the trunk. Stretching exercises can be performed by the patient or by a third party, such as the parents, physiotherapist, or doctor, with a frequency adapted to each individual case.

The device is available in two different sizes of the lengthening reserve, 50 mm or 80 mm, and it is as strong as the conventional rods to maintain the integrity of the construct, meaning it can be left in place permanently without the need for replacement or to perform arthrodesis at the end of growth.

**Figure 7:**
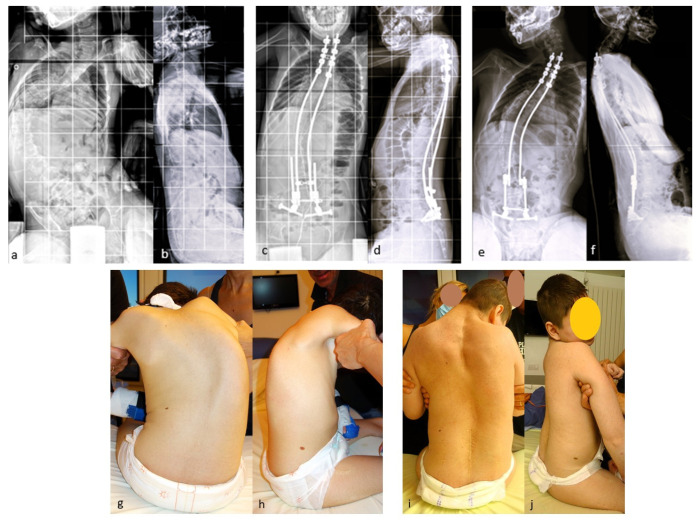
(**a**,**b**) Initial radiographs of a 12-year-old CP boy; (**c**,**d**) Immediate postoperative radiographs; (**e**,**f**) 5 year postoperative radiographs showing the expansion of the rods; (**g**–**j**) initial and 5 year postoperative clinical pictures.

A series of 23 patients with neuromuscular scoliosis was published in 2022 [32], demonstrating the benefits of these rods in patients with neuromuscular scoliosis (Figure 7).

The great advantage of these rods is that their elongation is gradual and spreads over time, which occurs when local conditions allow it. The lengthening is obtained owing to the viscoelastic relaxation of the soft tissues of the trunk, with less neurological risk, since it is obtained gradually in an awake patient.

The use of these self-expanding rods, which avoid repeated surgery, makes it possible to propose surgery at a younger age for progressive scoliosis in children. At this age, deformity is more flexible, allowing for better or even complete correction, which will then support the growth of the spine as an internal brace.

This new device can therefore reduce the overall cost of treating progressive scoliosis in children, improve the quality of life for young patients with scoliosis, and reduce the stress and psychological impact of repeated surgeries and hospitalizations.

A protocol is underway to compare the benefits of this self-expanding rod in patients with neuromuscular scoliosis with the Spring Distraction System, which is also a “growth-friendly” system [45]. 

Recently, self-expanding rods were implanted in 14 adolescent idiopathic scoliosis patients. The protocol has been published previously [46], but the results are not yet available. 

It should be noted that this work is not a systematic review, but we have been able to present the complete literature on the concept of the MIBF without selection bias. 

## 5. Future Directions

Many ongoing projects use this type of fixation. In our clinical experience, halo use is necessary. However, there is little formal evidence in the literature to determine the precise indications of this method in patients with neuromuscular scoliosis. 

We also questioned the extent of surgical indications for this technique. Projects are underway to evaluate its use in juvenile or early-onset idiopathic scoliosis. We also need to define its efficacy in a larger number of patients with constitutional bone diseases such as mucopolysaccharidosis. 

We are also looking at the question of surgical indications according to the age, weight, and severity of the deformity to ensure the best possible timing for surgery. There are still some gray areas in this respect. 

It may also be possible to use the concept of MIBF with other types of thoracic (e.g., pedicle screws) or pelvic fixation (e.g., S2-alar-iliac screws).

## 6. Key Recommendations

MIBF is a technically demanding method for the surgical treatment of spinal deformities in children and requires the following recommendations:

- To prepare rigid curves > 90° by preoperative halo-gravity traction for 4–6 weeks;
- To perform spinal fixation with solid bone anchorage at both ends of the construct to prevent mechanical complications such as implant pull-out and proximal junctional kyphosis;
- To obtain a sliding and effective construct owing to the progressive viscoelastic relaxation of the soft tissues obtained by rod lengthening;
- To preserve the intermediate area, which minimizes the early-onset of fibrosis and delays spontaneous bone fusion;
- Create a construct that is strong enough to avoid final fusion, thanks to the progressive stiffening of the spine over time;
- To achieve a less invasive surgical approach that preserves bone growth and allows surgery at a young age.

## 7. Conclusions

In conclusion, the concept of MIBF is the result of three decades of experience in fusionless surgery for early-onset scoliosis in children. The minimally invasive approach, satisfactory correction, stability over time, and ability to accommodate growth make it a complete and definitive treatment for neuromuscular scoliosis. Progressive ankyloses of the spine over time work as delayed arthrodesis. 

This treatment method is also particularly useful in the most difficult and complex spinal deformities, where higher-risk osteotomy or vertebral body resection techniques would be the other surgical options.

The use of the new self-expanding rod provides an additional advantage to the technique and a real benefit to the patient and their caregivers by avoiding repeated surgery. As a result, this device could save public health money by significantly reducing the complication rate and the overall cost of treating progressive scoliosis in children.

## Data Availability

The data presented in this study are available on request from the corresponding author. The data are not publicly available due to privacy concerns.
